# Environmental DNA (eDNA): A tool for quantifying the abundant but elusive round goby (*Neogobius melanostomus*)

**DOI:** 10.1371/journal.pone.0191720

**Published:** 2018-01-22

**Authors:** Meredith B. Nevers, Murulee N. Byappanahalli, Charles C. Morris, Dawn Shively, Kasia Przybyla-Kelly, Ashley M. Spoljaric, Joshua Dickey, Edward F. Roseman

**Affiliations:** 1 U.S. Geological Survey, Great Lakes Science Center, Lake Michigan Ecological Research Station, Chesterton, Indiana, United States of America; 2 National Park Service, Indiana Dunes National Lakeshore, Porter, Indiana, United States of America; 3 U.S. Geological Survey, Great Lakes Science Center, Ann Arbor, Michigan, United States of America; University of Hyogo, JAPAN

## Abstract

Environmental DNA (eDNA) is revolutionizing biodiversity monitoring, occupancy estimates, and real-time detections of invasive species. In the Great Lakes, the round goby (*Neogobius melanostomus*), an invasive benthic fish from the Black Sea, has spread to encompass all five lakes and many tributaries, outcompeting or consuming native species; however, estimates of round goby abundance are confounded by behavior and habitat preference, which impact reliable methods for estimating their population. By integrating eDNA into round goby monitoring, improved estimates of biomass may be obtainable. We conducted mesocosm experiments to estimate rates of goby DNA shedding and decay. Further, we compared eDNA with several methods of traditional field sampling to compare its use as an alternative/complementary monitoring method. Environmental DNA decay was comparable to other fish species, and first-order decay was lower at 12°C (k = 0.043) than at 19°C (k = 0.058). Round goby eDNA was routinely detected in known invaded sites of Lake Michigan and its tributaries (range log_10_ 4.8–6.2 CN/L), but not upstream of an artificial fish barrier. Traditional techniques (mark-recapture, seining, trapping) in Lakes Michigan and Huron resulted in fewer, more variable detections than eDNA, but trapping and eDNA were correlated (Pearson R = 0.87). Additional field testing will help correlate round goby abundance with eDNA, providing insight on its role as a prey fish and its impact on food webs.

## Introduction

The use of environmental DNA (eDNA) in ecological studies has the potential to revolutionize biodiversity monitoring, occupancy estimates, quantification of endangered and imperiled species, and real-time detections of invasive species spread [1–3]. Because DNA is shed by all living organisms, its detection and quantification in aquatic environments is being exploited as a surrogate measure of organisms’ presence [4]. This has the potential to eliminate the need to capture or visually detect a target species, making it a particularly attractive alternative as a stand-alone assay for organisms that are hard to capture, difficult to collect, or that have small populations. Worldwide, eDNA is being explored for multiple types of species and applications, from tree frogs in Trinidad [5] to crayfish in China [6]. Thus, integrating eDNA techniques with traditional monitoring has the potential to enhance greatly the quality and extent of natural resource monitoring programs.

The use of eDNA for detecting the spread of invasive species has expanded rapidly in recent years. Tools that are effective for early detection of invasive species are in high demand because evidence indicates that successful eradication is strongly linked to timely population control [7]. In the Great Lakes region of the USA, significant efforts are being directed to detecting the Asian carp (*Hypophthalmichthys* sp.) [8, 9], zebra and quagga mussels (*Dreissena polymorpha*, *Dreissena bugensis*) [10], and ruffe (*Gymnocephalus cernua*) [11].

The round goby (*Neogobius melanostomus*) is a benthic fish and highly prolific invader of freshwaters throughout the world [12, 13], originating from the Black and Caspian Seas. In the Great Lakes system, the round goby was first identified in 1990 in the St. Clair River [14]. Likely introduced from ballast water of ocean-going ships, the round goby spread rapidly throughout all five of the Great Lakes and into associated tributaries, largely as a result of anthropogenic activities [13]. It has become relatively abundant in four of the five Great Lakes [15] and has had multiple food web impacts. It continues to out-compete native benthic fishes for habitat and diet resources, including several sculpin and darter species (e.g., mottled sculpin, *Cottus bairdii*; slimy sculpin, *Cottus cognatus*; Johnny darter, *Etheostoma nigrum*) [16–19]. The round goby also preys upon fish eggs, which can potentially influence fish recruitment dynamics [20]. At the same time, some native predator fishes have shifted their diet to include the round goby [21], with implications for energy transfer and food web dynamics in both nearshore and offshore areas [22]. During warmer months, abundant populations thrive in breakwaters and in submerged vegetation of rivermouths [23], where larger individuals feed on a preferred diet of dreissenid mussels [13].

Despite the round goby’s increasing importance to multiple aspects of the Great Lakes food webs, reliable estimates of round goby abundance are confounded by behavior and habitat preference. In a review of capture methods, Johnson et al [24] concluded that a combination of bottom trawling and angling provided the best estimate of biomass, but neither was without its limitations: bottom trawling is limited by substrate type and is size-selective, and angling is somewhat biased to larger individuals. Round goby prefer hard substrate but can be found in a variety of habitats [13].

The biased underestimation of round goby abundance using traditional gears may be overcome through the use of eDNA. The method can be used in any substrate type and is not limited by fishing gear: all that is needed is a water sample. Before eDNA can be used as a round goby monitoring or quantification tool, however, an understanding of DNA release to the aqueous environment and the interactions of DNA with various biotic and abiotic factors must be understood. In this study, we use a quantitative PCR (qPCR) method in controlled mesocosm to determine DNA shedding rates and decay time and field studies to validate applications of the method. Species-specific studies were used to estimate quantity and variation among individuals in the laboratory and in the field, where environmental factors (e.g., temperature, turbidity, DNA settling) could influence eDNA results. We believe that the results from this study will be useful in advancing the science of round goby monitoring by achieving detection and biomass estimates of this invasive species using eDNA technology.

## Materials and methods

### Marker reliability

A qPCR assay previously developed by Nathan et al [25] was used in this study. For qPCR positive control, 6 individual round gobies collected with baited minnow traps from Thunder Bay, Lake Huron in August 2016 were frozen then shipped to the USGS aquatic laboratory in Chesterton, IN. Tissues were clipped from pectoral, dorsal, and tail fins with a sterile puncture, and DNA was extracted with DNeasy Blood and Tissue extraction kit (Qiagen, Germantown, MD) with overnight tissue lysis for 18 h at 56°C. DNA was extracted according to manufacturer’s instructions with one exception: the final DNA elution step included two sequential rinses of the column with 100 μL each of AE Buffer for a final volume of 200 μL. Tissue-derived DNA extracts were analyzed by the qPCR assay described below and confirmed high concentrations of targeted round goby marker.

### Quality assurance

To minimize DNA carryover and cross-contamination, all reusable items (i.e. collection bottles, filtration funnels, tweezers, beakers, peristaltic pump tubing) were cleaned and surface-sterilized between uses by soaking in 25%-50% bleach solution for at least 10 min and rinsing multiple times with autoclaved RO water; items were then autoclaved, once dry. Various sterile, single-use, disposable items (i.e., 50 ml pipettes, filtrations cups, sample collections cups) were used for sample collection and laboratory sample processing. Sample filtrations, DNA extractions, and qPCR assays were all performed in areas designated to be free from round goby DNA. Laboratory supplies and RO water were periodically tested for round goby DNA; no round goby eDNA was detected from any of these laboratory checks during the study.

### Mesocosom studies

**Fig 1:**
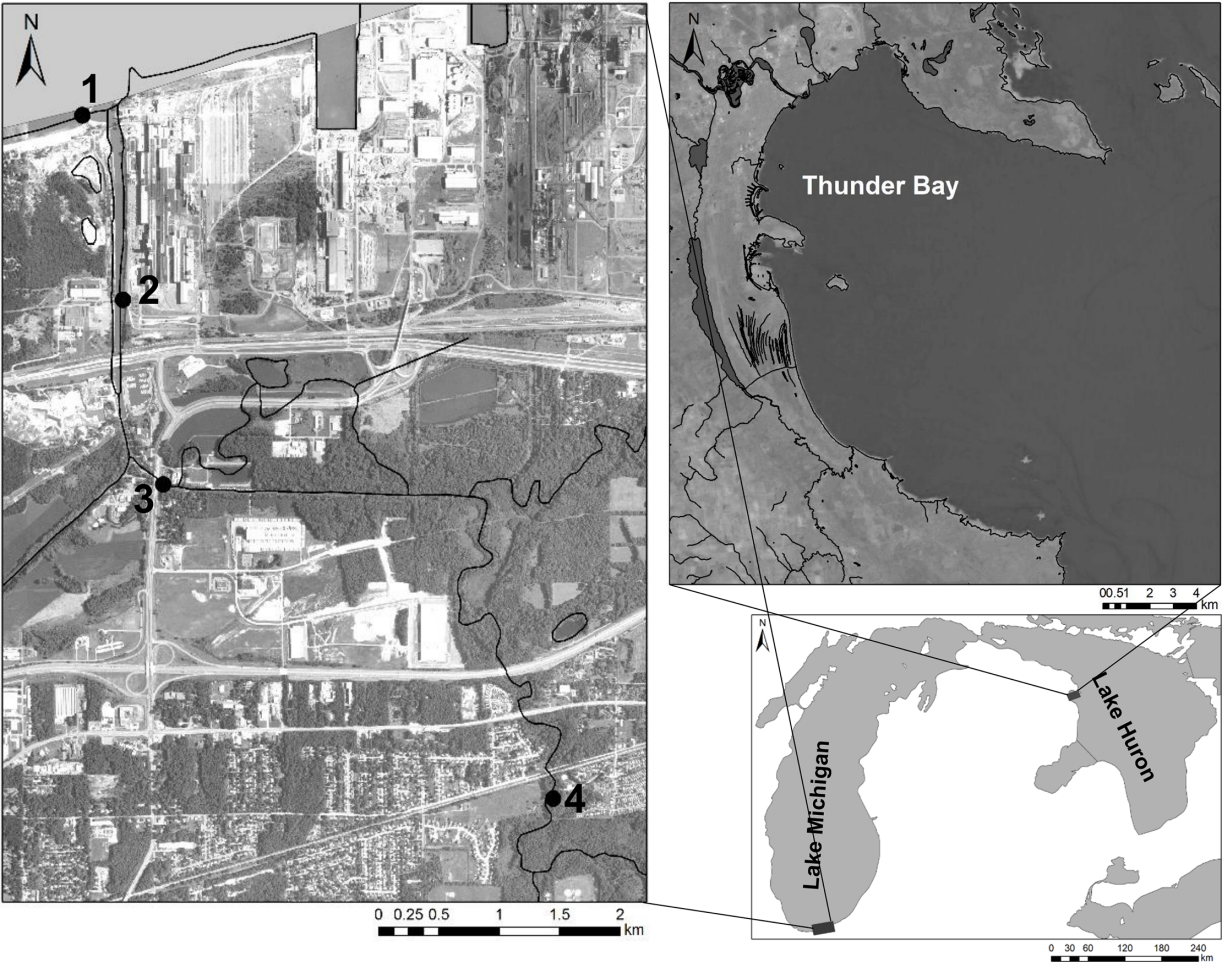
Map of the study areas in Lake Michigan and Lake Huron. Sampling sites in Lake Michigan (A) include the Portage Lakefront and Riverwalk breakwater (1), upstream sites (2 and 3), and a site above an elevation barrier (4). Sampling sites in Lake Huron (B) were located in Thunder Bay.

Mesocosm experiments were performed at the Indiana Dunes National Lakeshore (INDU) aquatic laboratory, in Porter, Indiana in February and March 2017 using round goby individuals obtained the previous fall. Using hook and line, size 14 egg hook, 2.61 kg monofilament line, round goby, 81–106 mm total length, 6.23–15.57 mg (n = 28), were obtained from Lake Michigan at the Portage Lakefront and Riverwalk Breakwater, INDU between November 1 and November 15, 2016 (Fig 1). The site borders a breakwater separating Burns Waterway from Lake Michigan and is known to harbor a large round goby population. The collected round goby were initially held in 113-liter aquaria chilled to match Lake Michigan ambient temperatures (12°C) and slowly acclimated to room temperature (21°C) over several days. Once acclimated, goby were transferred to a primary holding tank (custom made all-glass 227-liter aquarium) held at room temperature. Goby were transitioned from wild-caught isopods to commercially available pellet fish food over the following three months prior to beginning mesocosm experiments. Animal welfare in this study was approved by the National Park Service’s Institutional Animal Care and Use Committee (IACUC) and followed outlined protocols.

In an effort to create a mesocosm environment that was comparable with a natural ecosystem, with a diverse conglomeration of eDNA, water was used that was sourced from a 189-liter guppy aquarium maintained at room temperature. Guppies (~50) were obtained from a local pet store and fed commercially available flake fish food. This strategy incorporated biological activity (e.g., microbial) into the experiment that could potentially contribute to eDNA degradation due to predation and other mechanisms; it also increased the likelihood that eDNA of different fish species were present. The holding aquarium occupied by guppies (from here on referred as “source water” tank) was kept in a separate, round goby eDNA-free room to avoid contamination with goby eDNA from the experiment. Throughout the study period, the source tank water was repeatedly tested for the presence of round goby eDNA; all samples were negative. Three periods of mesocosm experiments were undertaken and designed to determine eDNA shedding dose response and temperature response, as well as to establish decay rates after round goby were removed (Table 1).

**Table 1:** Conditions for mesocosm experiments.

Mesocosm	Starting date	Experiment	Temperature (°C)	Round goby per tank	Goby Length (mm)	Goby weight (g)	Sampling intervals (hours)
A	2/1/2017	Shedding	19	1	93–116	9.38–13.52	T0, 3, 6, 12, 24
B	2/13/2017	Shedding	19	3	86–105	7.92–15.57	T0, 1, 2, 3, 6, 12, 24
C	2/14/2017	Decay	19	NA	NA	NA	T0, 6, 24, 48, 72, 168, 216, 312
D	3/20/2017	Shedding	12	3	86–106	6.8–15.23	T0, 3, 6, 12, 24
E	3/21/2017	Decay	12	NA	NA	NA	T0, 6, 24, 48, 72, 96, 144, 216, 312, 360

#### Experimental set-up

Each of the three periods of mesocosm experiments (inclusive of 3 shedding and 2 decay) included five, 9.5-liter, bleach-sterilized, glass aquaria filled with 7 liters of source water: three treatment tanks with gobies, one control tank with no gobies, and one tank of water for replacing volume in tanks after sample collection (S1 Fig; The individual in this manuscript has given written informed consent (as outlined in PLOS consent form) to publish these case details). In order to stabilize experimental conditions, tanks were filled with the source water two days prior to goby introduction. Clean airstones and tubes were installed for each individual tank, creating a closed system.

Two days prior to the start of the experiment, round goby were selected (3 or 9, depending on experiment, approximately the same size), weighed and measured, and placed in 10 cm x 10 cm plastic cages which were transferred into a goby eDNA-free 40L tank in order to acclimate to experimental conditions by slowly lowering the temperature to experimental conditions.

On the day of experimental set-up, individual cages were retrieved, round goby were removed with a nitrile-gloved hand, briefly dried (wicking away of excess water from the technician’s hand) on a clean towel, and released into an experimental tank. Tanks were then wrapped with plastic wrap to prevent cross-contamination between tanks and to minimize evaporation over time; an opening was cut in the plastic wrap with a sterile scalpel to allow sample collection.

All tanks were placed inside of an environmental chamber set with the following conditions: 19°C (experiment A, B, C) or 12°C (experiment D, E), 65% humidity, 12 h light:12 h dark photoperiod (Table 1). Two tanks were placed per shelf, spaced at least 30 cm apart.

Triplicate water samples for eDNA analysis (50 ml) were collected immediately following round goby placement into the experimental tanks and at regular intervals for each experiment. Prior to collecting samples, water was homogenized by gently stirring for ~20 sec with a sterile 50 mL pipette. Samples were then collected and each dispensed into individual sterile 90–120 ml polyethylene cups; a separate 50 ml-water sample was collected from each tank for turbidity and pH (experiments D and E) measurements. After each sample collection, water from the replacement water tank was added to each experimental and control tank to maintain volume at 7L. Sample cups were placed on ice, transported to USGS laboratory, and filtered within 0.5 h of collection.

#### Effect of number of individuals and temperature on eDNA shedding

Mesocosm experiments A (one round goby) and B (three round goby) were designed to determine rate of shedding with different numbers of individuals. Mesocosm experiment D (12°C) was designed to be compared to experiment B (19°C) to determine if temperature had an effect on eDNA shedding. Temperatures selected were within the range measured at the site of round goby collection between April and August (12–23°C). Shedding experiments took place over a 24 hour period (Table 1).

#### Decay rate

At the completion of each shedding experiment, round goby were removed from individual experimental tanks using dedicated, bleach-sterilized small nets in anticipation of using the tanks for eDNA decay experiments. The decay experiments (C and E) were implemented to determine the rate of DNA decay/degradation over time at two temperatures; experiments were initiated within 3 hours following the 24-hour sampling period for corresponding shedding mesocosms B and D without addition of replacement water after that collection. Similar to shedding experiments, triplicate sub-samples were collected at regular intervals (Table 1) with an additional 50-ml aliquot collected for water chemistry.

#### Quality control

For shedding experiments A, B, and D, water in the experimental tanks was analyzed for goby eDNA prior to the addition of gobies; all results were negative. Control tank water was tested at the same frequency as the three experimental aquaria throughout the course of each experiment; all samples were negative for goby eDNA, indicating no cross-contamination or carryover of eDNA between tanks. The growth chamber temperatures during the mesocosm experiments were maintained at 19°C for experiments A and B (measured range 19.7–20.3°C) and C (19.4–20.1°C) and at 12°C for experiments D and E (measured range 12.0–12.9°C).

### Field studies

To compare eDNA copy numbers with number of live round goby, two locations in the Laurentian Great Lakes were selected: western Lake Huron and southern Lake Michigan (Fig 1). In Thunder Bay, Lake Huron (45.0601°N, -83.390°W), a series of minnow traps made of wire mesh (42 cm long X 23 cm diameter, 2.5 cm opening at either end, 0.6 cm mesh) and attached to setlines was used to catch small, benthic fish, August 16–17, 2016. Traps were deployed in groups of 3 at 4 locations near artificial reefs and at a depth of 14 feet. Traps were baited with either worms or cheese and set for 14–19 hours. Prior to trap retrieval, a Niskin sampler was deployed to collect a surface water sample and a bottom water sample just above the traps. The Niskin sampler was deployed in an open position and rinsed with surface lake water between samples and sampling events to minimize eDNA carryover. Water samples (n = 8) were poured into sterile, DNA-free 1L size polypropylene bottles and kept in a cooler on ice; samples were transported to USGS laboratory in Chesterton, IN and filtered within 24h of collection onto 1.5 μm glass fiber filters. Upon trap retrieval, fish species present were identified, counted, and recorded.

In southern Lake Michigan at Portage Lakefront and Riverwalk (41.632°N, -87.179°W), Indiana Dunes National Lakeshore (Fig 1A, site 1), a mark-recapture survey was undertaken starting September 7, 2016. Prior to initiating fishing activity, lake water samples were collected for eDNA analysis along a designated 10-m stretch of the breakwater at three equidistant points. Water depth was approximately 1.5–2m deep. A horizontal sampler (Wildco, Yulee, Florida) was deployed to collect a surface water sample and a bottom water sample. The horizontal sampler was lowered in an open position to the designated depth and triggered. Water samples (n = 6) were poured into sterile, DNA-free 1L size polypropylene bottles and kept in a cooler on ice; samples were transported to USGS laboratory and filtered within 4h of collection onto 1.5 μm glass fiber filters.

Following eDNA water sample collection, round goby were caught using a hook and line for a total of 120 minutes; one individual used size 14 egg hooks and 2.61 kg monofilament line. Captured gobies were held in a floating live-well to minimize mortality until fishing was complete. Before releasing the captured goby back to the sample collection area, each fish had the distal margin of its right pectoral fin clipped using scissors, thus providing a distinct mark to be identified in future collections. After 6 days, hook and line capture was repeated in the same location, and recaptured round goby, those with the distinctive fin clip, were counted and recorded.

#### Seasonal eDNA collection

A survey of round goby eDNA was conducted during the ice-free period from August 2016-April 2017 at the breakwater associated with the Portage Lakefront and Riverwalk (Fig 1A). Water samples were collected in triplicate from just off the lake bottom using a peristaltic pump (Geotech Environmental Equipment, Inc.) or horizontal bottom sampler (Wildco, Yulee, Florida) on five occasions in August, November, March, and April (n = 16). Pump tubing was sterilized in the laboratory by passing it through 2L of 25% bleach solution followed by rinsing with at least 4L of autoclaved RO water. In the field, samples for eDNA were collected by pumping the water just above the sediment surface into 1L size bleach-sterilized polypropylene bottles; tubing was changed between samples. When using horizontal bottom sampler, the device was cleaned with 95% ethanol and rinsed with RO water between sites. Alternately, water was directly filtered using a modified collection pole; a sterile, disposable filtration cup fitted with a 1.5um glass-fiber filter was affixed to the end of telescopic 10 ft long sampling pole and water was pumped through the tubing into a collection bucket until 2L was recorded (S2 Fig). Additional water samples (n = 15) were collected in a survey upstream in the Burns Waterway/Little Calumet River in July 2017 to determine if round goby eDNA could be detected in the river water (Fig 1A, sites 2 and 3) and upstream of an elevation barrier to fish passage (Fig 1A, site 4); round goby have not been identified upstream of the barrier, to our knowledge.

### Water chemistry and physical parameters

At the time of eDNA field sampling, dissolved oxygen and water temperature (ProODO, YSI, Inc, Yellow Springs, OH) were tested. In the laboratory, mesocosm samples were analyzed for turbidity (2100N turbidimeter, Hach, Loveland, Colorado) and for the 12°C mesocosm study, samples also were analyzed for pH (Thermo Fisher Scientific, Inc, Waltham, MA).

### Sample processing and DNA extraction

Water samples (1000 or 2000 mL) for eDNA studies were filtered through 1.5-μm glass microfiber filters (GE Healthcare Life Sciences, Pittsburgh, PA) following Nathan et al. (2015) and mesocosm tank water samples (50 mL) were filtered through 0.22-μm nitrocellulose filters (EMD Millipore, Billerica, MA). Filters were placed in Mo Bio PowerWater® bead tubes (MO BIO Laboratories, Inc., Carlsbad, CA) and held at -20°C until processing. DNA was extracted directly from the filters using Mo Bio PowerWater® kit according to manufacturer’s instructions with one exception: the final DNA elution step included two sequential rinses of the column with 50 μL each of PW6 for a final volume of 100 μL. DNA concentration for all samples was measured by fluorometric quantification (Qubit® High Sensitivity dsDNA HS Assay, Thermo Fisher Scientific, Waltham, MA), and DNA quality was measured using Nanophotometer 260/280 ratio (Nanophotometer Pearl, Implen Inc, Westlake Village, CA).

### Quantitative real-time PCR (qPCR) assays

Samples were analyzed in triplicate (technical replicates) for round goby using the following primer and probe set: GobyCOI-F2*d*: 5’- CTTCTGGCCTCCTCTGGTGTTG-3’, GobyCOI-R2d: 5’-CCCTAGAATTGAGGAAATGCCGG-3’, and GobyCOI-Pr: 5’-6-FAM-CAGGCAACTTGGCACATGCAG-BHQ-3’ [25]. qPCR assays were performed using the Bio-Rad CFX Connect™ Real-time PCR Detection System (Bio-Rad, Hercules, California) in clear 96 well PCR plates. Each plate was sealed by heat bonding (AccuSeal PS1000, Labnet International, Edison, New Jersey) and contained three no-template controls to assess contamination. Each 20 uL reaction contained: 10 uL of TaqMan Environmental Master Mix 2.0 (ThermoFisher Scientific), 1.8 uL of each 10-μM primer (forward and reverse), 0.05 uL of 100-μM hydrolysis probe, 2 μL template DNA, and 4.35 uL of PCR-grade water. Thermocyler conditions were as follows: 95°C for 10 min, and 40 cycles of 95°C for 15 s and 60°C for 1 min.

Quantification of samples was determined from standard curves obtained from six ten-fold serial dilutions of a gBlock® Gene Fragment (Integrated DNA Technologies, Coralville, Iowa). The gBlock® was synthesized from a 149 bp DNA fragment obtained from an alignment of sixty-nine *Neogobius melanostomus* COI sequences obtained from the GenBank database (https://www.ncbi.nlm.nih.gov/). The sequences were aligned using MEGA version 6 [26], the area from which the primer and probe set was developed was located, and the 149 bp fragment encompassed this area. The gBlock® Gene Fragment concentration was determined using the NanoPhotometer Pearl. Results are reported as copy numbers (CN)/1 L.

For each assay, amplification efficiency and R^2^ was determined; standard curves for all runs had an R^2^ ≥ 0.99, and amplification efficiency ranged between 89–101%. Two instances occurred during qPCR analysis of mesocosm experiments B and C, in which the quantification of standard curve dilutions were less than expected, a result that affects sample quantification. In order to minimize time and resources, 15 samples were rerun in duplicate to ensure Cq values were similar and unaffected. Results showed that Cq values were unaffected, and therefore an average standard curve was created from five different curves that were generated during qPCR analysis for mesocosm experiments B and C. The average standard curve had an acceptable amplification efficiency (97%) and R^2^ (0.996) and thus was applied to the Cq values from the original runs and CN was calculated. The lower limit of quantification (LLOQ) was established by averaging the Cq values obtained from the highest dilutions from standard curves across all qPCR runs in which at least 2 of the 3 technical replicates were detected. This resulted in a LLOQ of Cq = 38 (corresponding to an average of 16 CN/rx), which was subsequently used to determine if a sample Cq value falls within the Range of Quantification (ROQ).

Samples were considered positive if the Cq value was within the ROQ and also if samples were detected above the LLOQ; samples were considered negative (non-detect; ND) if there was no exponential curve crossing the threshold value before cycle 40. If the triplicate reactions resulted in incomplete outcome (ROQ value for 1 or 2 replicates and non-detect for 1 or 2 replicates), the original samples were reanalyzed in triplicate per Goldberg et al. [27]. The average quantities of the second round of amplification were used if the outcome was complete (combination of ROQ and DNQ or DNQ combined with ND). If the outcome on the second round was still incomplete, the sample was rerun for a third time, and the average quantities of the third round of amplification were used. Samples in which the CN was above the LLOQ were still considered positive and were reassigned a quantity of half of the LLOQ (CN/rx = 8) [28] and samples that were considered negative (non-detect) were assigned a value of zero; all data were averaged across technical replicates for use in statistical analysis.

### Statistical analysis

Statistical analyses and graphical representations were performed using Systat version 13 [29], SPSS version 23 [30], Primer 7 [31], and R version 3.3.1 [32]. Pearson correlation analysis was used to determine significant relationships between number of round goby and eDNA concentrations and between round goby eDNA concentrations and measured physical factors. Mixed effects models, with tanks (mean of tank replicates) as a random effect and using the lme4 package in R, were used to determine if temperature, 19°C versus 12°C; number of round goby, 1 versus 3; and round goby weight, mesocosm B versus D had an effect on eDNA concentration (copy numbers (CN)/L). The natural log of CN was used in all mixed effects models; however, anti-log values are reported for consistency. Ranges and means for CN/L are averaged across field/mesocosm and/or technical (qPCR wells) replicates. Significance was tested with a Likelihood Ratio Test using the ANOVA function in R. The Likelihood Ratio Test compares the goodness of fit of two models, the null model (without fixed effect) and the full model (with fixed effect). Principal Components Analysis with a maximum of three components was used to compare concentrations of eDNA over time with physical conditions; data were normalized prior to analysis, and highest eigenvectors (coefficients) are presented.

#### Decay

Round goby eDNA decay rates were calculated using a first-order decay model for each temperature mesocosm (C and E). There were few instances in which a zero (below detection limit) was recorded for the eDNA concentration. The limit of detection was not established for this study, therefore, in order to incorporate those observations in the estimation of k, the zeroes were replaced with an empiric detection limit/2 or ½ the quantity of the minimum positive DNA concentration measured. An exponential decay rate was calculated following previous decay models [33, 34]:

$$ln(C/C_{0})=-kt$$ (1)

Allowing the model to estimate decay rate and the initial concentration, we use the equation:

$$\ln\left(C\right)=\ln\left(C_{0}\right)-kt$$ (2)

Where C = the concentration of eDNA in CN/L, C_0_ is the initial eDNA concentration, t = time since the start of experiment in hours, k = first order decay rate constant.

A mixed effects model that includes tank as a random factor was used to calculate k and C_0_ using the lme4 package in R. The resulting slope of the line and intercept provided the first-order decay function, k, and the initial eDNA concentration, C_0_, respectively.

Half-life for DNA survival was calculated by incorporating C_t_ = $\frac{1}{2}$C_0_ into Eq (2):

$$t_{\frac{1}{2}}={log}_{1-k}\;\frac{1}{2}$$ (3)

#### Shedding

In order to calculate shedding rate, eDNA concentration must reach steady state: when eDNA shedding is in equilibrium with eDNA decay. Shedding was calculated after eDNA concentration had reached steady state. Because not all tank replicates reached steady state, averages were based only on those that achieved this status. All shedding rates were calculated as net shedding, which incorporated eDNA loss to decay, using k as described above.

$$V\frac{dC}{dt}=S-kCV$$ (4)

Where V is the volume (L) of the tanks, C is the eDNA concentration (CN/L), t is time (hrs), S is the eDNA shedding rate (CN/hr), and k is the calculated first order decay rate constant/hr. At steady state, $\frac{dC}{dt}=0\;and\;S=kCV$

Decay rates from mesocosm C were assumed for calculating shedding for mesocosm A. Shedding rates were also calculated per number of individual round goby and per g of round goby.

## Results

### Mesocosm: eDNA shedding

**Fig 2:**
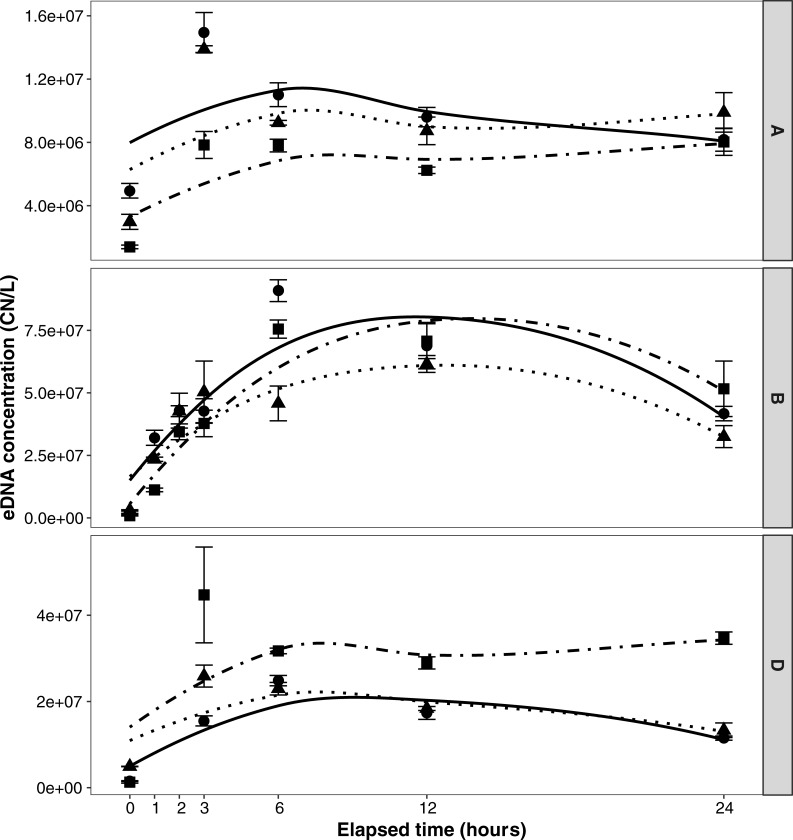
Shedding of eDNA concentrations over time (Loess smoothing method) for mesocosm experiments. (A) one round goby individual, 19°C; (B) three round goby individuals, 19°C; and (D) three round goby individuals, 12°C. Symbols/lines represent tank replicates as follows: tank 1, circle/solid; tank 2, triangle/dotted; and tank 3, square/dot-dash.

Pattern of shedding was similar among all three mesocosm experiments (A, B, D), with a high concentration of round goby eDNA detected at T_0_ (~1–2 min after goby addition). This was most dramatic in mesocosm B, where initial concentration was 10^6^ CN/L and increased to more than 10^7^ CN/L in the first hour (Fig 2). In all three mesocosms, concentration increased over the first several hours, peaking around hour 3–6. Variation was higher overall in mesocosm B.

#### Number of gobies and temperature effects on DNA shedding

According to a likelihood ratio test the number of round goby (1 versus 3 individuals, mesocosm A vs. B) had a significant effect on eDNA concentration over time (χ^2^ (1) = 7.5054, p = 0.0062); according to the mixed effects model the mesocosm B (3 gobies) had approximately 3.12 CN/L ± 1.51 (model coefficient) more than mesocosm A (1 round goby). The weight differences of the 3 gobies in mesocosm B versus D did not have a significant impact on eDNA concentration over time (χ^2^ (1) = 0.152, p = 0.70).

According to a likelihood ratio test, temperature (12 versus 19°C, mesocosm B vs. D) did not significantly affect eDNA concentration over time (χ^2^ (1) = 2.2901, p = 0.1302); however, according to the mixed effects model, eDNA concentration in mesocosm D (12°C) were approximately 0.53 CN/L ± 1.55 (model coefficient) lower than those in mesocosm B (19°). High variation was observed.

#### Shedding rate

Steady state was reached after hour 3 in two of the replicate tanks of mesocosm A and in one of the replicate tanks of mesocosm D. Steady state was not attained in the other tanks, likely due to length of experiment or the sampling interval, so calculations are based only on those that achieved steady state. For mesocosm D, decay rate (k) from mesocosm E was used, and a mean shedding rate was calculated as 9.5x10^6^ CN/hr, 3.2x10^6^ CN/hr/goby, and 2.5x10^5^ CN/hr/g round goby. For mesocosm A, decay rate (k) was assumed from mesocosm C to calculate shedding rate and a mean shedding rate was calculated as 3.4x10^6^ CN/hr and 3.0x10^5^ CN/hr/g round goby.

### Mesocosm: eDNA decay

**Fig 3:**
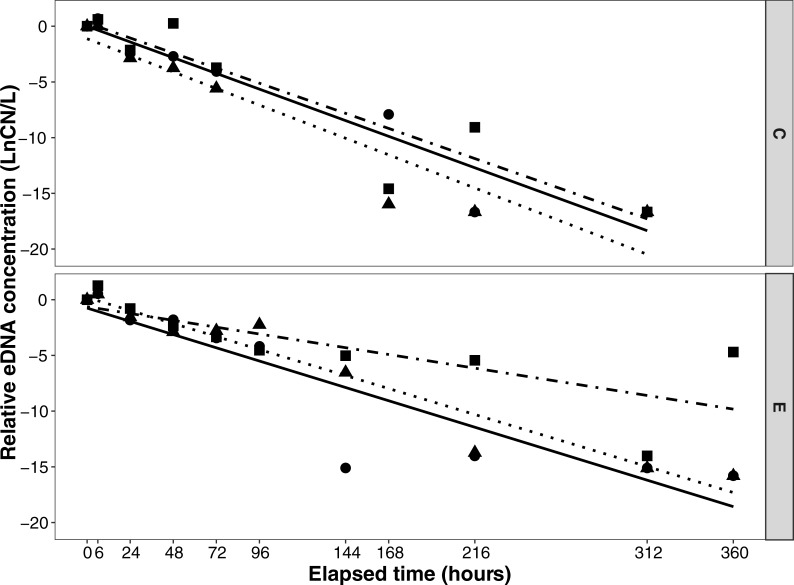
Natural log of relative eDNA concentration (CN/CN_0_) over time for mesocosm decay. Mesocosm experiments were conducted at two temperatures: C (19°C) and E (12°C). Lines signify linear model (lm) regression method (R, ggplot2). Symbols/lines represent tank replicates as follows: tank 1, circle/solid; tank 2, triangle/dotted; and tank 3, square/dot-dash.

First order decay constants (± standard error) were calculated as k = 0.058 (0.0049) in mesocosm C (19°C) and k = 0.043 (0.0044) in mesocosm E (12°C) (Fig 3). The mesocosm E decay rate was lower than that of the mesocosm C (t = 1.82, p = 0.14).

Decreases in round goby eDNA concentration followed an exponential curve for mesocosms C and E. eDNA non-detect was reached between 7 and 9 days in mesocosm C (19°C) and between 13 and 15 days in mesocosm E (12°C), indicating more rapid degradation at warmer temperatures. There was one aberrant result in mesocosm E, where one replicate tank had quantifiable eDNA at day 15 despite a non-detect on day 13. Variation in eDNA concentration between replicate tanks increased as values approached zero, particularly in mesocosm E, with range between replicates maximized at 21,421 CN/L 6 days after the experiment was initiated.

Half-life estimations for eDNA were shorter for the 19°C mesocosm: estimates were 11.68 hours for mesocosm C (19°C) and 15.85 hours for mesocosm E (12°C).

### Field: Number of round goby vs. eDNA detection

Comparison of round goby detection using baited traps in Lake Huron and mark-recapture in Lake Michigan resulted in regular detection of the eDNA marker. Individual traps captured between 0 and 7 round goby. Trap gangs (3 traps/gang) had a total of 0–11 round goby. Incidental rock bass (*Ambloplites rupestris*) and log perch (*Percina caprodes*) were also trapped (2 individuals each). Goby target eDNA concentration in water samples ranged 0–1,332 CN/L for samples collected at the surface and 0–3,756 CN/L for samples just above the lake bottom (14m water depth). In mark-recapture, a total of 33 individuals were caught initially and marked. In the second visit, 23 individuals were collected, and of these 6 were marked; a total for the reach was calculated as 127 round goby. Goby target eDNA concentrations for samples ranged 0–5,070 CN/L for surface water samples and 6,121–6,757 CN/L for samples collected just above the lake bottom (1.5 m water depth).

**Fig 4:**
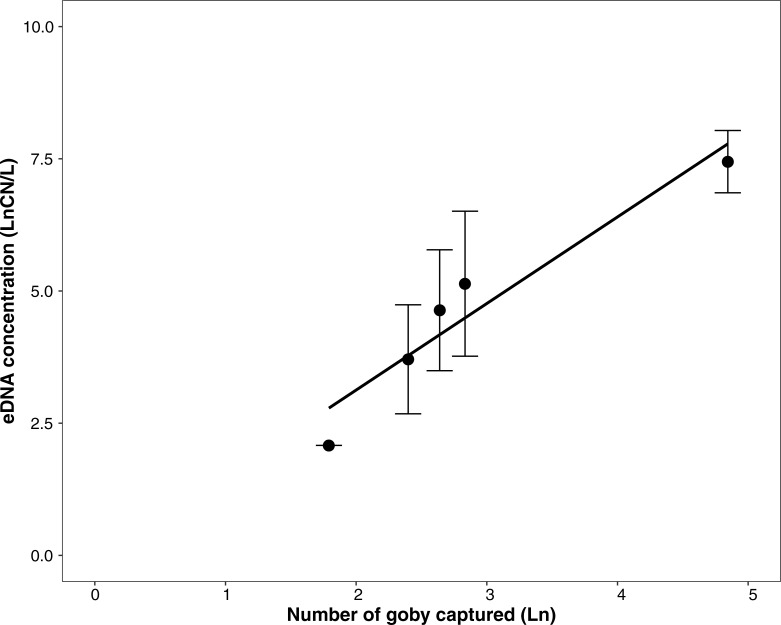
Relationship between and number of round goby captured (natural log, LN) and concentration of round goby eDNA detected (natural log, LN), with best-fit regression line (Pearson R = 0.871).

Because there was no significant difference with depth of sample collection (t = 0.335, p = 0.747), consistent with Hinlo et al [35], concentrations for surface and bottom water at a given site were averaged for correlation analysis. Number of round goby collected and eDNA were correlated across samples (trap set or mark-recapture) (N = 5, Pearson R = 0.871) (Fig 4).

On a separate sampling occasion, eDNA was sampled at locations upstream and downstream of an elevation barrier (Fig 1). Goby eDNA was regularly detected at two downstream locations on two separate occasions, with concentration ranging from 3,414–19,541 CN/L. No round goby eDNA was detected upstream of the barrier.

### Field: Influence of physical factors on eDNA detection

**Fig 5:**
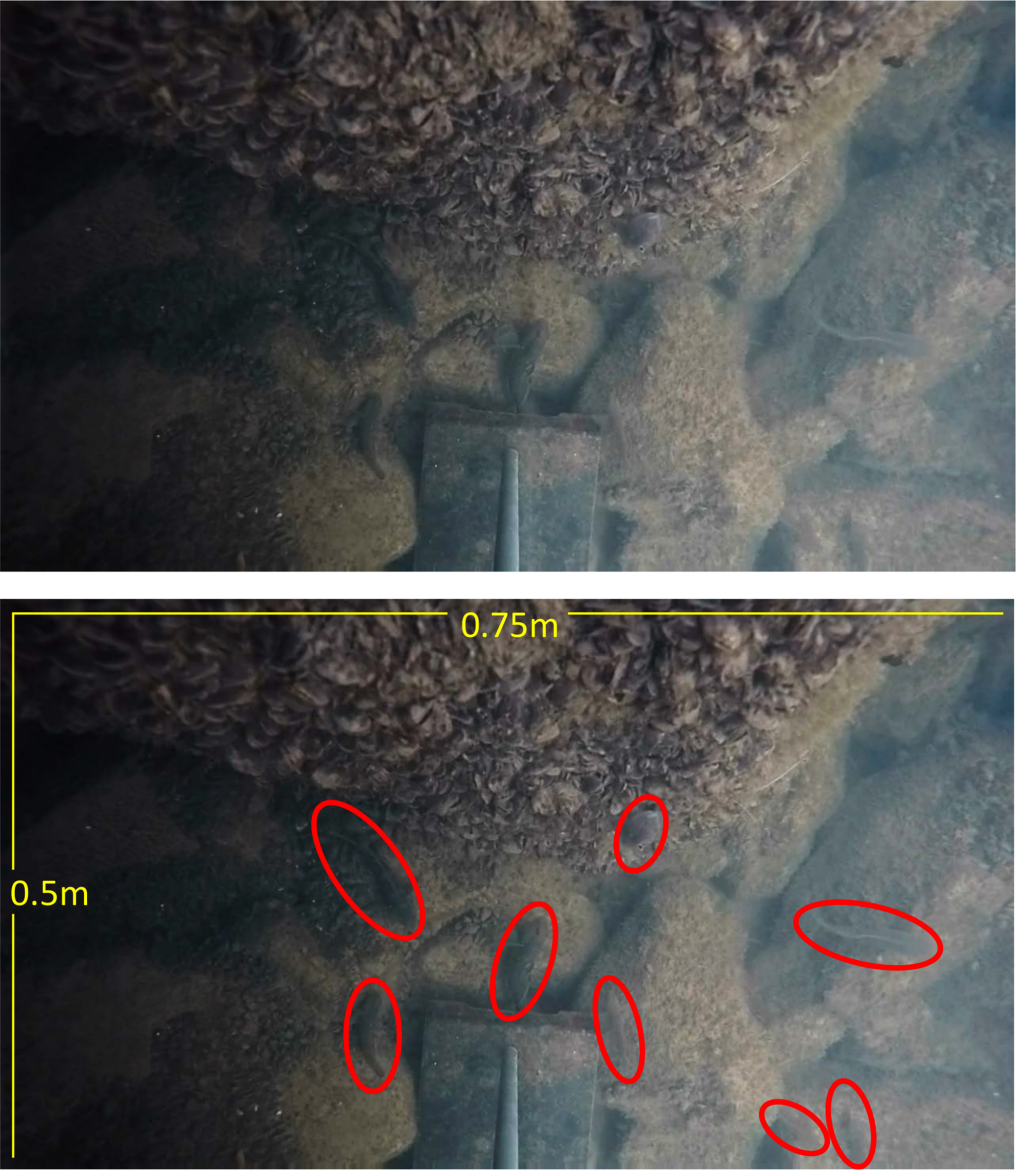
Captured still photo (GoPRO, Inc., San Mateo, California) of round goby at Portage Lakefront Breakwater, November 1, 2016 (A). Individuals highlighted with circles (B). Note extensive coverage by Dreissenid mussels. Depth = 1.5 m.

A survey of eDNA concentrations resulted in consistent detection of round goby eDNA through the ice-free period. The site of sample collection has a large population of round goby (Fig 5) due to abundant available breakwater habitat and the warming and nutrient influence of a large river.

Concentration of eDNA was positively correlated with month of sample collection (Pearson R = 0.623, N = 5) and water temperature (R = 0.411) and eDNA was negatively correlated with pH (R = -0.578). In a principal component’s analysis, component 1 was best explained by a combination of month, water temperature (range 5.1–23.8°C), and dissolved oxygen concentration (7.69–11.01 mg/L), but eigenvectors were not particularly high (-0.466, -0.433, 0.446). Component 2 increased with dissolved oxygen (0.535).

## Discussion

The use of eDNA for detection of aquatic organisms is expanding rapidly and applications are being enthusiastically sought by researchers and natural resource managers hoping to conserve endangered and imperiled species [36, 37], identify populations of cryptic species [38], distinguish subpopulations [39], and stem the tide of invasive species [6, 10], worldwide. With its genesis and development in the realm of microbial ecology, eDNA technology faces a suite of new challenges when applied to the eukaryotic domain.

### eDNA shedding

The release of DNA from aquatic organisms can be through sloughing, defecation, gamete release, or organism demise. Its subsequent interactions and environmental fate are not well understood. Overall shedding rate for the round goby in our mesocosm studies was calculated after reaching steady state. It was assumed that the rapid initial increase in eDNA was the results of physiological stress due to transfer between tanks, which has been noted in other studies [28]. Several replicates did not reach steady-state, indicating that perhaps future studies should be extended beyond 24 hours. As expected, there was a higher overall amount of eDNA measured in the tanks with three individuals than in the tanks with one individual, but the relationship was directly related, with shedding rates averaging between 2.5 and 3.2 X 10^5^ CN/h/g fish. Under our experimental conditions, the presence of additional gobies did not appear to impact an individual goby’s shedding rate: the highest shedding rate was measured in a tank holding only one goby.

In our experiments, we purposely selected round goby individuals that fell within a given size/age class (ranging 9.38–13.6 g) in an attempt to normalize release rates. Other studies have identified significant differences in age classes, with juvenile bluegill (*Lepomis macrochirus*) generally having a higher rate of eDNA release [40] and larger individual carp (*Cyprinus carpio*, *Hypophthalmichthys nobilis*, *Hypophthalmichthys molitrix*) associated with higher eDNA releases [28, 41]. In order to quantify populations using eDNA, a better understanding of multiple biological factors affecting release is needed, including age class, interspecies interactions [42], food availability, and reproductive status, as well as DNA stability. In one of our mesocosm tanks, a significant egg release was observed, but there was no associated spike in eDNA detection. Research by Bylemans et al. [43] showed that spawning activity of the Macquarie perch (*Macquaria australasica*) resulted in an increase in nuclear DNA but not mitochondrial DNA—the primary target of our qPCR assay. The gobies used in the experiment were not sexed prior to the experiment, so both males and females were presumably used. Further studies should include a comparison of shedding rates between male and female fish and also the usefulness of this information for measuring spawning activity.

Temperature did not have a significant influence on shedding of our round gobies. Specimens were collected during late summer near a rocky breakwater bordering a large river; ambient temperature was measured as 23.3°C. Takahara et al. [41] similarly found no impact of water temperature on eDNA shedding in common carp (*Cyprinus carpio*), having tested temperatures of 7, 15, and 25°C, and neither did Klymus [28], testing freshwater carp to temperatures as high as 31°C. In a tropical tilapia species (*Oreochromis mossambicus*), however, experiments in 23, 29, and 35°C resulted in higher eDNA shedding at higher temperatures [44]. Thermal tolerance of the target species could impact the rate at which a stress response occurs, with tropical tilapia tolerating much higher temperatures than a temperate freshwater invasive. Round goby can survive a broad range of temperatures, but generally prefer warmer temperatures, with energetic optimum recorded as 26°C [45]. Maximizing a monitoring assay for round goby should consider the range of temperatures under which sampling would be conducted and also whether extreme stress, and therefore the potential for increased eDNA shedding, would be expected.

### eDNA decay

eDNA decay rates are critical to understanding the usefulness of eDNA and other genomic monitoring tools for field applications because measurements need to detect and quantify *recent* occupancy by a species. Concerns about widespread applicability of eDNA for monitoring have centered on the persistence of eDNA after release and the effect of environmental conditions on decay [46, 47]. Several studies have examined eDNA decay in a number of fish [40, 42, 48] and amphibian [49] species, and eDNA generally behaves conservatively with a relatively rapid decay in the natural environment. Most studies have focused on decay in an aqueous environment, and questions about differences in eDNA integrity in sediment-associated particles remain; some studies indicate longer survival in stream sediment [47].

In our mesocosm study, calculated first-order decay constants for round goby (k = 0.043–0.058) were slightly lower than those reported by Sassoubre et al [42] for several marine fish species, including northern anchovy (*Engraulis mordax*), Pacific sardine (*Sardinops sagax*) and Pacific Chub Mackerel (*Scomber japonicas*) (k = 0.055–0.101) and by Maruyama et al. [40] for the freshwater bluegill sunfish (*Lepomis macrochirus*) (k = 0.051–0.159) (S1 Table). Round goby eDNA declined steadily in our mesocosms following the removal of the fish, and after 7–15 days, eDNA was no longer detectable in the system under our experimental conditions. Reports for non-detection range widely across mesocosm experiments: 1–2 days for three-spined stickleback (*Gasteroseus aculeatus*) and 7 days for European flounder (*Platichthys flesus*) [34] and 14 days for Siberian sturgeon (*Acipenser baerii*) [50]. See also Eichmiller et al. [51].

eDNA decay was slightly lower in cold water than warm water in our mesocosm. We found that eDNA was detected for a longer period of time (15 days) in mesocosm E (12°C) than in mesocosm C (19°C). Because round goby survive in such a wide range of temperatures, perhaps the difference in our experiment of (12 and 19°C) was not sufficient to cause a significant difference. Pilliod et al. [52] detected salamander eDNA after 18 days in a controlled experiment held at 4°C with no light but non-detection was observed much sooner at higher temperatures. More rapid eDNA degradation for *Cyprinus carpio* and ayu (*Plecoglossus altivelis altivelis*) was found by Tsuji et al. [53], whose findings also rejected the hypothesis implicating bacterial growth with degradation at higher temperatures. Other researchers found no impact of temperature on eDNA decay rates [28, 44]. Rapid decay at higher temperatures may be attributed to synergistic effects, with temperature interacting with other variables.

In addition to water temperature, there are multiple abiotic factors that might influence the decay and degradation of eDNA in natural waters. We measured both turbidity and pH through shedding and decay studies but did not manipulate these as study variables; both turbidity and pH were stable over the length of the study. Studies targeting eDNA have found that UV-B, and sunlight exposure may increase the rate of eDNA decay [1, 46]. UV has long been harnessed for microbial disinfection of bacteria in drinking and wastewater for its ability to damage bacterial DNA [54–56]; unlike extracellular eDNA, culturable microbes (e.g., bacteria) have the ability to repair damaged DNA within the cell.

Half-life for the two mesocosms ranged from 12–15 hours, indicating a fairly rapid decline in eDNA detection. This is somewhat longer than results reported by Maruyama [40]: 6.3 h. The result confirms eDNA as a conservative monitoring method and the indication of recent presence of a species, a key characteristic of an effective detection tool.

### Field detection

Detection of eDNA was consistent in locations where round goby was captured or known to be present, in Lakes Michigan and Huron and in the Little Calumet River (Fig 1). Further, eDNA was not detected in the location upstream of an artificial barrier, which gives credence to its conservative nature in field detection. Of particular importance is the positive correlation between concentration of eDNA and number of round goby collected, using two different capture techniques. While the sample size was somewhat limited, multiple studies have found similar results with other fish species [41, 42]. While a combination of angling and trawling has been identified as the most efficient and cost-effective, [24] bottom type and was not suitable for trawling, so we chose to use angling and trapping. Ideally, visual inspection through the use of scuba divers or cameras would likely provide the most complete data but can be cost-limited. Future work will expand on some preliminary visual counting tools (Fig 5).

Variation in the eDNA signal is still in the early stages of discovery, but in locations with high concentrations of the target species, it appears that the signal is correlative to population size. Other life history factors that influence the signal could include range of water column occupied [12] or type of habitat being sampled (e.g., lentic/lotic, size, and flow). Research by O’Donnell et al. [57] has puzzled out spatial variation in the eDNA signal for metazoans in nearshore marine waters and confirms that even in the dynamic environment, eDNA was conserved spatially and detection was limited to a measured population.

Among the physical characteristics, water temperature had the greatest influence on eDNA copy numbers, a finding that could be related to seasonal cycles of the round goby population, including spawning and migration. Other factors included month and dissolved oxygen, both of which could relate to seasonality as well. Correlative data on round goby numbers would help distinguish the relationship between number of individuals, concentration of eDNA, and physical characteristics and whether seasonality is related to round goby populations or survival and detection of the eDNA signal.

### Future steps

The use of eDNA holds great promise for revolutionizing our approach to species monitoring and management, providing a conservative and conservation-considered method that can improve our spatial and temporal coverage of detection. Results presented here provide another advance toward applying the technique for quantification of a target organism, the invasive round goby. While eDNA techniques are still evolving, there is considerable demand for their use by natural resource management agencies. One area where eDNA application gains momentum is in integrating this technology with traditional fishery surveys [58] and the potential to replace labor-intensive surveys in monitoring programs. Further, combining eDNA technology with other genomic techniques (e.g., metabarcoding, whole-genome sequencing) could help management agencies study multiple populations in a single survey.

As eDNA applications expand, information gaps are revealed that need to be addressed before the technology can be fully integrated into monitoring programs and embraced by research managers. A great deal of scholarship in microbial ecology has been devoted to detection of microbial species in field settings use DNA-based technology, and this history can be used to inform our understanding of eDNA survival and transport in natural environments. One of the key differences for eDNA species detection is differentiation between intracellular and extracellular DNA. Where microbial surveys detect the entire bacterial cell and therefore primarily intracellular DNA, eukaryotic eDNA may include both intracellular and extracellular material [42], which can affect estimates of occupation, temporally, and rates of degradation [59].

Once eDNA is shed into the aqueous environment, it is subject to biological, chemical, and physical processes that all interact to account for an instantaneous concentration measurement collected in a water sample. As demonstrated in this study, conditions of the water (i.e., temperature, dissolved oxygen) influence concentration and detection. Further, hydrodynamic influences of settling, resuspension, and dispersion can impact eDNA concentration as well. Attention to all of the components of a conceptual model [42, 60, 61] for eDNA will help inform our understanding of what is being collected and relay it back to species quantification.

The highly successful invasive round goby has spread to all of the Great Lakes and continues to invade and spread through associated tributaries. Because of its potential influence on the lake food web as it displaces native prey fish, confident estimates of its population size and biomass are needed. Results presented here show that the round goby DNA is shed and decays at a conservative rate and that it can be regularly detected in areas inhabited by round goby. These results present a realistic application of eDNA for estimating round goby presence and abundance, findings that can be used in early-warning monitoring of species spread and to expand applications of eDNA for fishery sciences.

## Supporting information

S1 Fig Mesocosm design. (A) Experimental tanks were bleach sterilized, rinsed, air dried, and filled with 7 L source water. (B) Round goby used in mesocosm experiments were acclimated prior to placement in experimental tanks. (C and D) After acclimation, round goby were placed in experimental tanks and tanks were subsequently plastic wrapped. (E) Experimental tanks were placed in the diurnal growth chamber set to desired conditions. (F) Samples were taken in triplicate at specified times.
(PDF)

S2 Fig Sample collection design. (A) Sterile, disposable cup fitted with a glass-fiber filter (1.5um) was attached to a telescoping sampling pole, with tubing was strung along the pole to a peristaltic pump. (B) Sterile cup was placed directly into the water. (C) With this design, only the cup had to be replaced between samples and the pole/exterior of tube rinsed in 10% bleach solution. Filtered water was emptied into graded bucket to keep track of volume filtered. (D) In the field, the filter was removed using sterile technique and (E) placed in an extraction tube; all samples were placed on ice until return to the laboratory.
(PDF)

S1 Table Published decay rates for eDNA for a variety of different aquatic organisms. (PDF)

## References

[1] Barnes MA, Turner CR, Jerde CL, Renshaw MA, Chadderton WL, Lodge DM. Environmental conditions influence eDNA persistence in aquatic systems. Environmental Science and Technology. 2014;48(3):1819–27. doi: 10.1021/es404734p 24422450 10.1021/es404734p

[2] Goldberg CS, Turner CR, Deiner K, Klymus KE, Thomsen PF, Murphy MA, et al Critical considerations for the application of environmental DNA methods to detect aquatic species. Methods in Ecology and Evolution. 2016;7(11):1299–307. doi: 10.1111/2041-210X.12595

[3] Lodge DM, Turner CR, Jerde CL, Barnes MA, Chadderton L, Egan SP, et al Conservation in a cup of water: Estimating biodiversity and population abundance from environmental DNA. Mol Ecol. 2012;21(11):2555–8. doi: 10.1111/j.1365-294X.2012.05600.x 22624944 10.1111/j.1365-294X.2012.05600.x PMC3412215

[4] Ficetola GF, Miaud C, Pompanon F, Taberlet P. Species detection using environmental DNA from water samples. Biol Lett. 2008;4(4):423–5. doi: 10.1098/rsbl.2008.0118 18400683 10.1098/rsbl.2008.0118 PMC2610135

[5] Brozio S, Manson C, Gourevitch E, Burns TJ, Greener MS, Downie JR, et al Development and application of an eDNA method to detect the critically endangered Trinidad golden tree frog (*Phytotriades auratus*) in bromeliad phytotelmata. PLoS ONE. 2017;12(2). doi: 10.1371/journal.pone.0170619 28199338 10.1371/journal.pone.0170619 PMC5310848

[6] Cai W, Ma Z, Yang C, Wang L, Wang W, Zhao G, et al Using eDNA to detect the distribution and density of invasive crayfish in the HongheHani rice terrace World Heritage site. PLoS ONE. 2017;12(5). doi: 10.1371/journal.pone.0177724 28505200 10.1371/journal.pone.0177724 PMC5432173

[7] N'Guyen A, Hirsch PE, Bozzuto C, Adrian-Kalchhauser I, Horkova K, Burkhardt-Holm P. A dynamical model for invasive round goby populations reveals efficient and effective management options. J Appl Ecol. 2017;2017:1–11. doi: 10.1111/1365-2664.12934

[8] Amberg JJ, Grace McCalla S, Monroe E, Lance R, Baerwaldt K, Gaikowski MP. Improving efficiency and reliability of environmental DNA analysis for silver carp. J Great Lakes Res. 2015;41(2):367–73. doi: 10.1016/j.jglr.2015.02.009

[9] Jerde CL, Chadderton WL, Mahon AR, Renshaw MA, Corush J, Budny ML, et al Detection of Asian carp DNA as part of a Great Lakes basin-wide surveillance program. Can J Fish Aquat Sci. 2013;70(4):522–6. doi: 10.1139/cjfas-2012-0478

[10] Egan SP, Grey E, Olds B, Feder JL, Ruggiero ST, Tanner CE, et al Rapid molecular detection of invasive species in ballast and harbor water by integrating environmental DNA and light transmission spectroscopy. Environmental Science and Technology. 2015;49(7):4113–21. doi: 10.1021/es5058659 25686279 10.1021/es5058659

[11] Tucker AJ, Chadderton WL, Jerde CL, Renshaw MA, Uy K, Gantz C, et al A sensitive environmental DNA (eDNA) assay leads to new insights on Ruffe (*Gymnocephalus cernua*) spread in North America. Biol Invasions. 2016;18(11):3205–22. doi: 10.1007/s10530-016-1209-z

[12] Adrian-Kalchhauser I, Burkhardt-Holm P. An eDNA assay to monitor a globally invasive fish species from flowing freshwater. PLoS ONE. 2016;11(1). doi: 10.1371/journal.pone.0147558 26814998 10.1371/journal.pone.0147558 PMC4729461

[13] Kornis MS, Mercado-Silva N, vander Zanden MJ. Twenty years of invasion: A review of round goby *Neogobius melanostomus* biology, spread and ecological implications. J Fish Biol. 2012;80(2):235–85. doi: 10.1111/j.1095-8649.2011.03157.x 22268429 10.1111/j.1095-8649.2011.03157.x

[14] Jude DJ, Reider RH, Smith GR. Establishment of Gobiidae in the Great Lakes basin. Can J Fish Aquat Sci. 1992;49(2):416–21.

[15] Bunnell DB, Barbiero RP, Ludsin SA, Madenjian CP, Warren GJ, Dolan DM, et al Changing ecosystem dynamics in the Laurentian Great Lakes: Bottom-up and top-down regulation. Bioscience. 2014;64(1):26–39. doi: 10.1093/biosci/bit001

[16] Bergstrom M, Mesinger A. Interspecific resource competition between the invasive round goby and three native species: longperch, slimy sculpin, and spoonhead sculpin. Trans Am Fish Soc. 2009;138:1009–17.

[17] Corkum LD, Sapota MR, Skora KE. The round goby, *Neogobius melanostomus*, a fish invader on both sides of the Atlantic Ocean. Biol Invasions. 2004;6(2):173–81. doi: 10.1023/B:BINV.0000022136.43502.db

[18] French JRP, III, Jude DJ. Diets and diet overlap of nonindigenous gobies and small benthic native fishes co-inhabiting the St. Clair River, Michigan. J Great Lakes Res. 2001;27(3):300–11.

[19] Lauer TE, Allen PJ, McComish TS. Changes in mottled sculpin and johnny darter trawl catches after the appearance of round gobies in the Indiana waters of Lake Michigan. Trans Am Fish Soc. 2004;133(1):185–9. doi: 10.1577/T02-123

[20] Chotkowski MA, Marsden JE. Round goby and mottled sculpin predation on lake trout eggs and fry: Field predictions from laboratory experiments. J Great Lakes Res. 1999;25(1):26–35.

[21] Madenjian CP, Tapanian MA, Witzel LD, Einhouse DW, Pothoven SA, Whitford HL. Evidence for predatory control of the invasive round goby. Biol Invasions. 2011;13:987–1002.

[22] Madenjian CP, Stapanian MA, Witzel LD, Einhouse DW, Pothoven SA, Whitford HL. Evidence for predatory control of the invasive round goby. Biol Invasions. 2011;13(4):987–1002. doi: 10.1007/s10530-010-9884-7

[23] Cooper MJ, Ruetz CR, III, Uzarski DG, Shafer BM. Habitat use and diet of the round goby (*Neogobius melanostomus*) in coastal areas of lake michigan and lake huron. J Freshwat Ecol. 2009;24(3):477–88. doi: 10.1080/02705060.2009.9664321

[24] Johnson TB, Allen M, Corkum LD, Lee VA. Comparison of methods needed to estimate population size of round gobies (*Neogobius melanostomus*) in western Lake Erie. J Great Lakes Res. 2005;31:78–86.

[25] Nathan LR, Jerde CL, Budny ML, Mahon AR. The use of environmental DNA in invasive species surveillance of the Great Lakes commercial bait trade. Conserv Biol. 2014;29(2):430–9. doi: 10.1111/cobi.12381 25169113 10.1111/cobi.12381

[26] Tamura K, Stecher G, Peterson D, Filipski A, Kumar S. MEGA6: Molecular evolutionary genetics analysis version 6.0. Mol Biol Evol. 2013;30(12):2725–9. doi: 10.1093/molbev/mst197 24132122 10.1093/molbev/mst197 PMC3840312

[27] Goldberg CS, Sepulveda A, Ray A, Baumgardt J, Waits LP. Environmental DNA as a new method for early detection of New Zealand mudsnails (*Potamopyrgus antipodarum*). Freshwater Science. 2013;32(3):792–800. doi: 10.1899/13-046.1

[28] Klymus KE, Richter CA, Chapman DC, Paukert C. Quantification of eDNA shedding rates from invasive bighead carp *Hypophthalmichthys nobilis* and silver carp *Hypophthalmichthys molitrix*. Biol Conserv. 2015;183:77–84. doi: 10.1016/j.biocon.2014.11.020

[29] SYSTAT. Systat 13.0. Chicago, IL: SYSTAT Software, Inc.; 2009.

[30] SPSS. SPSS, Version 23. IBM Corp, SPSS Inc., Armonk, NY; 2014.

[31] Primer-E Ltd. Plymouth Routines In Multivariate Ecological Research (Primer) 7. Primer 7010. Auckland, New Zealand2015.

[32] R Core Team. R: A language and environment for statistical computing. In: R Foundation for Statistical Computing, editor. Vienna, Austria 2017.

[33] Schnell IB, Thomsen PF, Wilkinson N, Rasmussen M, Jensen LRD, Willerslev E, et al Screening mammal biodiversity using DNA from leeches. Curr Biol. 2012;22(8):R262–R3. doi: 10.1016/j.cub.2012.02.058 22537625 10.1016/j.cub.2012.02.058

[34] Thomsen PF, Kielgast J, Iversen LL, Møller PR, Rasmussen M, Willerslev E. Detection of a diverse marine fish fauna using environmental DNA from seawater samples. PLoS ONE. 2012;7(8). doi: 10.1371/journal.pone.0041732 22952584 10.1371/journal.pone.0041732 PMC3430657

[35] Hinlo R, Furlan E, Suitor L, Gleeson D. Environmental DNA monitoring and management of invasive fish: Comparison of eDNA and fyke netting. Management of Biological Invasions. 2017;8(1):89–100. doi: 10.3391/mbi.2017.8.1.09

[36] Bohmann K, Evans A, Gilbert MTP, Carvalho GR, Creer S, Knapp M, et al Environmental DNA for wildlife biology and biodiversity monitoring. Trends in Ecology and Evolution. 2014;29(6):358–67. doi: 10.1016/j.tree.2014.04.003 24821515 10.1016/j.tree.2014.04.003

[37] Jerde CL, Mahon AR, Chadderton WL, Lodge DM. "Sight-unseen" detection of rare aquatic species using environmental DNA. Conservation Letters. 2011;4(2):150–7. doi: 10.1111/j.1755-263X.2010.00158.x

[38] Uchii K, Doi H, Minamoto T. A novel environmental DNA approach to quantify the cryptic invasion of non-native genotypes. Molecular Ecology Resources. 2016;16(2):415–22. doi: 10.1111/1755-0998.12460 26307935 10.1111/1755-0998.12460

[39] Darling JA. Genetic studies of aquatic biological invasions: closing the gap between research and management. Biol Invasions. 2015;17(3):951–71. doi: 10.1007/s10530-014-0726-x

[40] Maruyama A, Nakamura K, Yamanaka H, Kondoh M, Minamoto T. The release rate of environmental DNA from juvenile and adult fish. PLoS ONE. 2014;9(12). doi: 10.1371/journal.pone.0114639 25479160 10.1371/journal.pone.0114639 PMC4257714

[41] Takahara T, Minamoto T, Yamanaka H, Doi H, Kawabata Z. Estimation of fish biomass using environmental DNA. PLoS ONE. 2012;7(4). doi: 10.1371/journal.pone.0035868 22563411 10.1371/journal.pone.0035868 PMC3338542

[42] Sassoubre LM, Yamahara KM, Gardner LD, Block BA, Boehm AB. Quantification of Environmental DNA (eDNA) Shedding and Decay Rates for Three Marine Fish. Environmental Science and Technology. 2016;50(19):10456–64. doi: 10.1021/acs.est.6b03114 27580258 10.1021/acs.est.6b03114

[43] Bylemans J, Furlan EM, Hardy CM, McGuffie P, Lintermans M, Gleeson DM. An environmental DNA-based method for monitoring spawning activity: a case study, using the endangered Macquarie perch (*Macquaria australasica*). Methods in Ecology and Evolution. 2017;8(5):646–55. doi: 10.1111/2041-210X.12709

[44] Robson HLA, Noble TH, Saunders RJ, Robson SKA, Burrows DW, Jerry DR. Fine-tuning for the tropics: application of eDNA technology for invasive fish detection in tropical freshwater ecosystems. Molecular Ecology Resources. 2016;16(4):922–32. doi: 10.1111/1755-0998.12505 26849294 10.1111/1755-0998.12505

[45] Lee VA, Johnson TB. Development of a bioenergetics model for the round goby (*Neogobius melanostomus*). J Great Lakes Res. 2005;31(2):125–34.

[46] Strickler KM, Fremier AK, Goldberg CS. Quantifying effects of UV-B, temperature, and pH on eDNA degradation in aquatic microcosms. Biol Conserv. 2015;183:85–92. doi: 10.1016/j.biocon.2014.11.038

[47] Turner CR, Uy KL, Everhart RC. Fish environmental DNA is more concentrated in aquatic sediments than surface water. Biol Conserv. 2015;183:93–102. doi: 10.1016/j.biocon.2014.11.017

[48] Eichmiller JJ, Bajer PG, Sorensen PW. The relationship between the distribution of common carp and their environmental DNA in a small lake. PLoS ONE. 2014;9(11). doi: 10.1371/journal.pone.0112611 25383965 10.1371/journal.pone.0112611 PMC4226586

[49] Pilliod DS, Goldberg CS, Arkle RS, Waits LP. Estimating occupancy and abundance of stream amphibians using environmental DNA from filtered water samples. Can J Fish Aquat Sci. 2013;70(8):1123–30. doi: 10.1139/cjfas-2013-0047

[50] Dejean T, Valentini A, Duparc A, Pellier-Cuit S, Pompanon F, Taberlet P, et al Persistence of environmental DNA in freshwater ecosystems. PLoS ONE. 2011;6(8). doi: 10.1371/journal.pone.0023398 21858099 10.1371/journal.pone.0023398 PMC3152572

[51] Eichmiller JJ, Best SE, Sorensen PW. Effects of Temperature and Trophic State on Degradation of Environmental DNA in Lake Water. Environmental Science and Technology. 2016;50(4):1859–67. doi: 10.1021/acs.est.5b05672 26771292 10.1021/acs.est.5b05672

[52] Pilliod DS, Goldberg CS, Arkle RS, Waits LP. Factors influencing detection of eDNA from a stream-dwelling amphibian. Molecular Ecology Resources. 2014;14(1):109–16. doi: 10.1111/1755-0998.12159 24034561 10.1111/1755-0998.12159

[53] Tsuji S, Ushio M, Sakurai S, Minamoto T, Yamanaka H. Water temperature-dependent degradation of environmental DNA and its relation to bacterial abundance. PLoS ONE. 2017;12(4). doi: 10.1371/journal.pone.0176608 28448613 10.1371/journal.pone.0176608 PMC5407774

[54] Ki SJ, Ensari S, Kim JH. Solar and tidal modulations of fecal indicator bacteria in coastal waters at Huntington Beach, California. Environ Manage. 2007;39(6):867–75. doi: 10.1007/s00267-006-0154-5 17453279 10.1007/s00267-006-0154-5

[55] Süß J, Volz S, Obst U, Schwartz T. Application of a molecular biology concept for the detection of DNA damage and repair during UV disinfection. Water Res. 2009;43(15):3705–16. doi: 10.1016/j.watres.2009.05.048 19576614 10.1016/j.watres.2009.05.048

[56] Whitman RL, Nevers MB, Korinek GC, Byappanahalli MN. Solar and temporal effects on *Escherichia coli* concentration at a Great Lakes swimming beach. Appl Environ Microbiol. 2004;70(7):4276–85. doi: 10.1128/AEM.70.7.4276-4285.2004 15240311 10.1128/AEM.70.7.4276-4285.2004 PMC444827

[57] O'Donnell JL, Kelly RP, Shelton AO, Samhouri JF, Lowell NC, Williams GD. Spatial distribution of environmental DNA in a nearshore marine habitat. PeerJ. 2017;2017(2). doi: 10.7717/peerj.3044 10.7717/peerj.3044 PMC5333549 28265513

[58] Thomsen PF, Møller PR, Sigsgaard EE, Knudsen SW, Jørgensen OA, Willerslev E. Environmental DNA from seawater samples correlate with trawl catches of subarctic, deepwater fishes. PLoS ONE. 2016;11(11). doi: 10.1371/journal.pone.0165252 27851757 10.1371/journal.pone.0165252 PMC5112899

[59] Dell'Anno A, Corinaldesi C. Degradation and turnover of extracellular DNA in marine sediments: Ecological and methodological considerations. Appl Environ Microbiol. 2004;70(7):4384–6. doi: 10.1128/AEM.70.7.4384-4386.2004 15240325 10.1128/AEM.70.7.4384-4386.2004 PMC444808

[60] Barnes MA, Turner CR. The ecology of environmental DNA and implications for conservation genetics. Conserv Genet. 2016;17(1):1–17. doi: 10.1007/s10592-015-0775-4

[61] Nevers MB, Boehm AB. Modeling fate and transport of fecal bacteria in surface water In: Sadowsky MJ, Whitman RL, editors. The Fecal Bacteria. Washington, DC: ASM Press; 2011 p. 165–88.

