# Supplementary figures and images for: Environmental DNA (eDNA): A tool for quantifying the abundant but elusive round goby (Neogobius melanostomus)
Source: PLoS One. 2018 Jan 22;13(1):e0191720. doi: 10.1371/journal.pone.0191720 (PMC5777661; doi:10.1371/journal.pone.0191720)

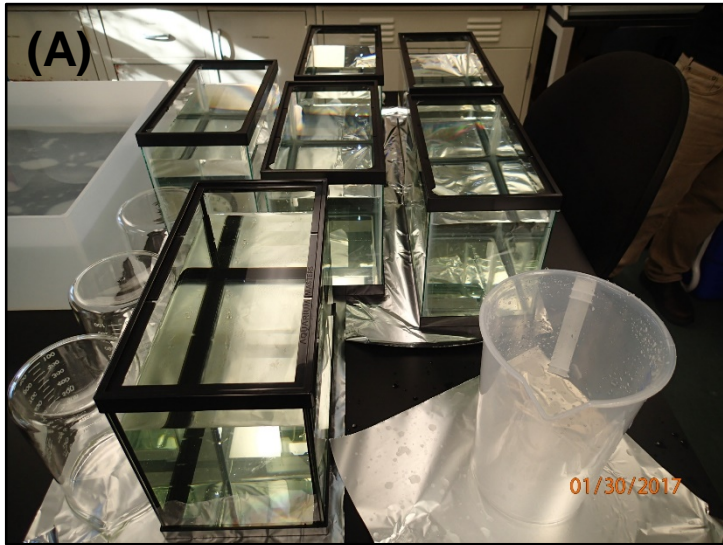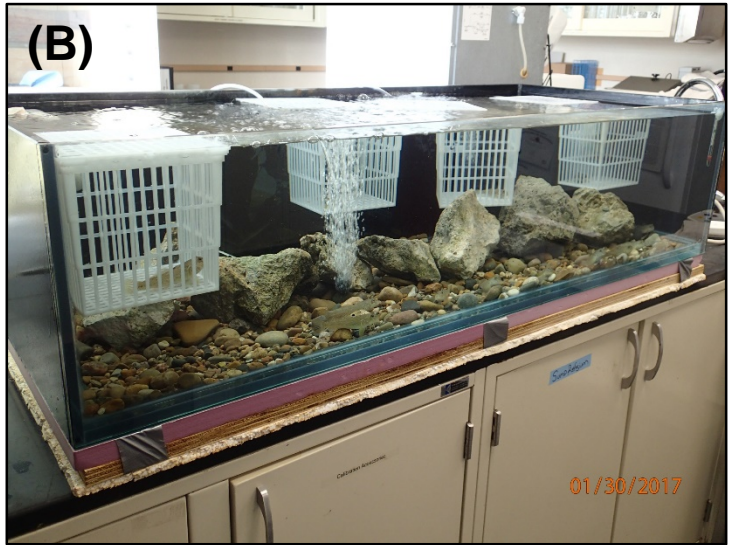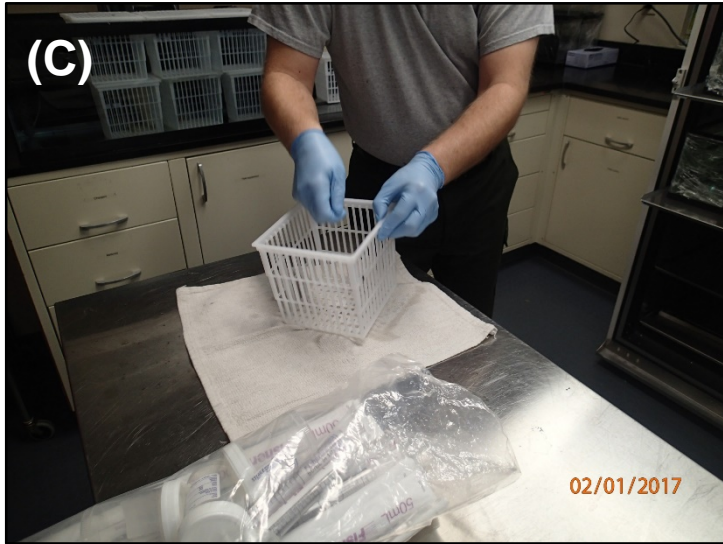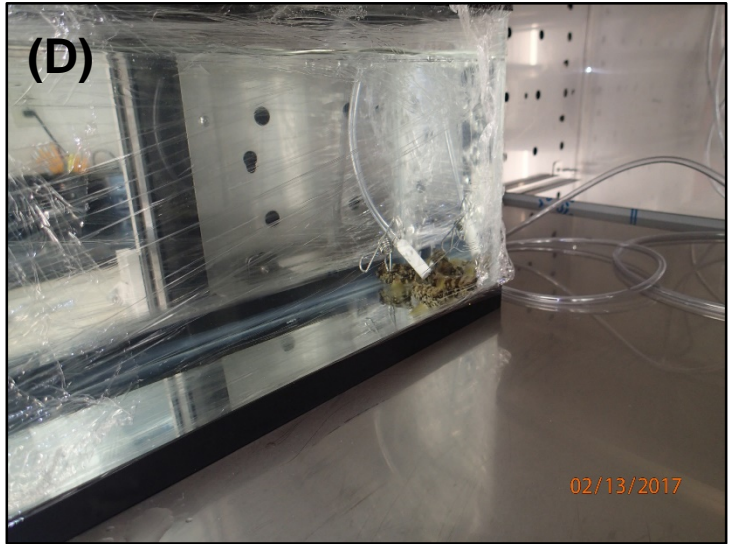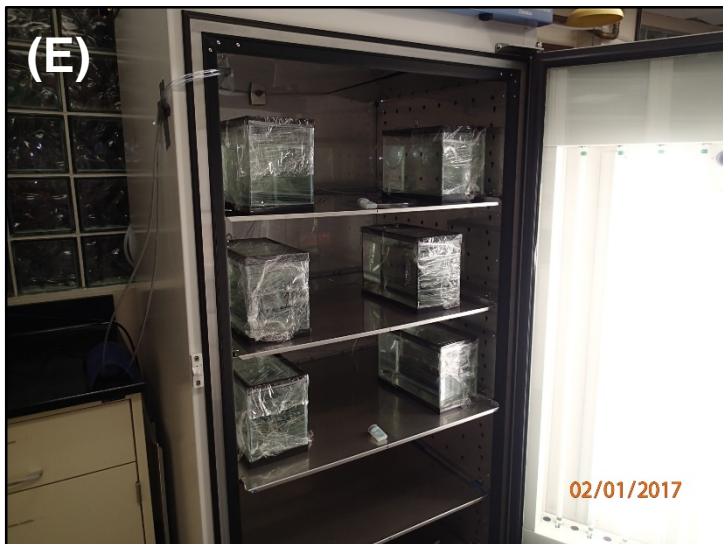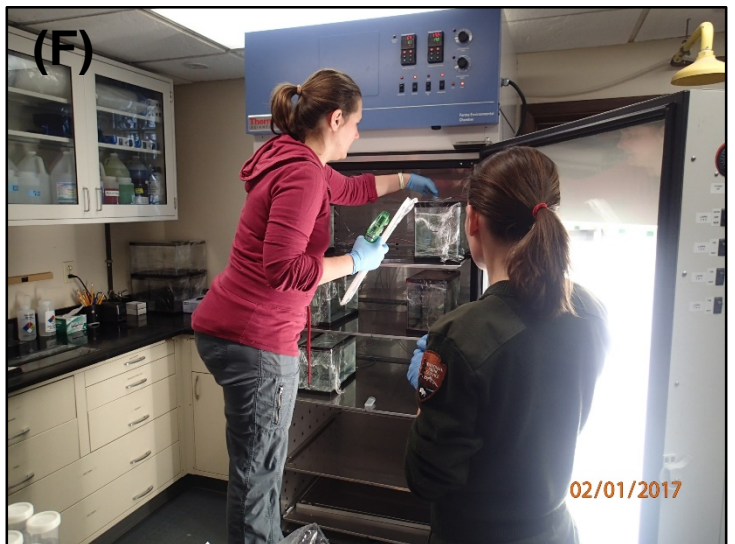

Supplement: S1 Fig — (A) Experimental tanks were bleach sterilized, rinsed, air dried, and filled with 7 L source water. (B) Round goby used in mesocosm experiments were acclimated prior to placement in experimental tanks. (C and D) After acclimation, round goby were placed in experimental tanks and tanks were subsequently plastic wrapped. (E) Experimental tanks were placed in the diurnal growth chamber set to desired conditions. (F) Samples were taken in triplicate at specified times. (PDF) [file pone.0191720.s001.pdf]

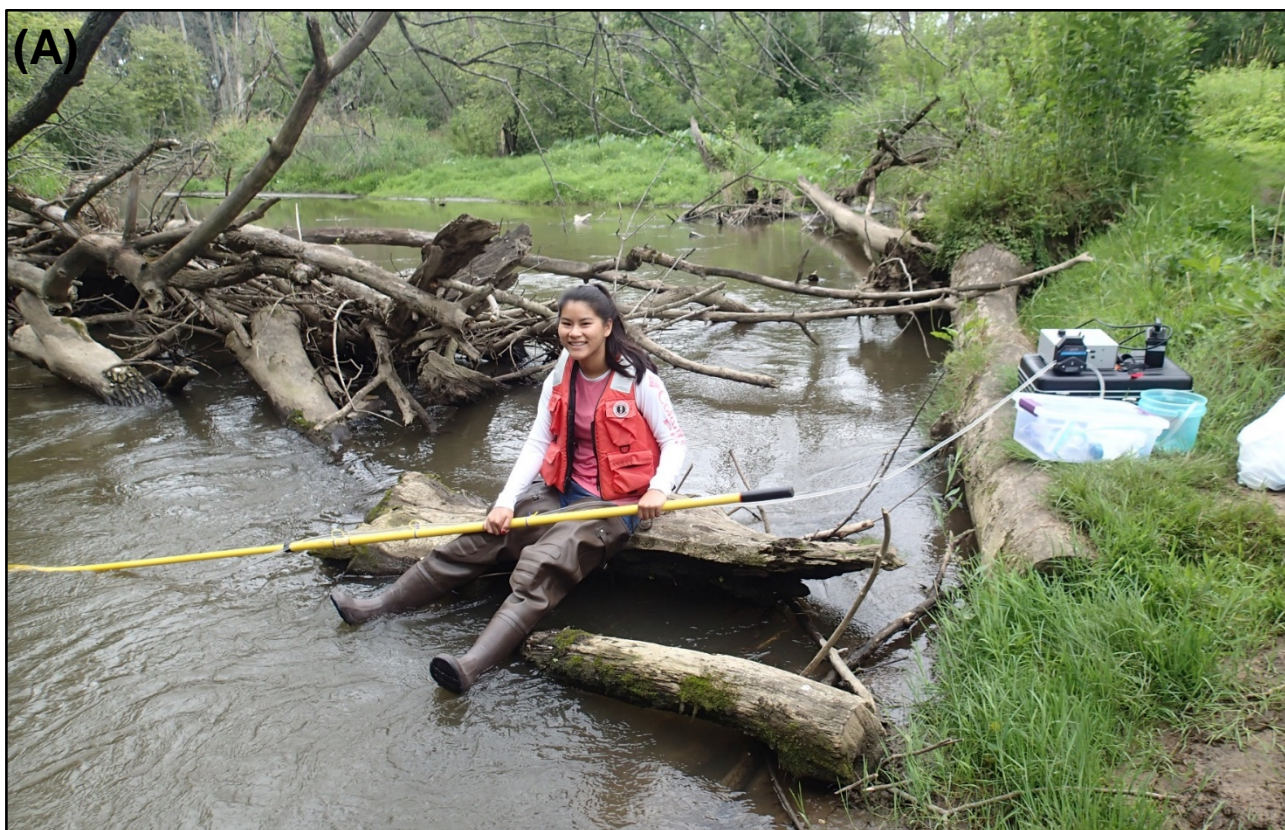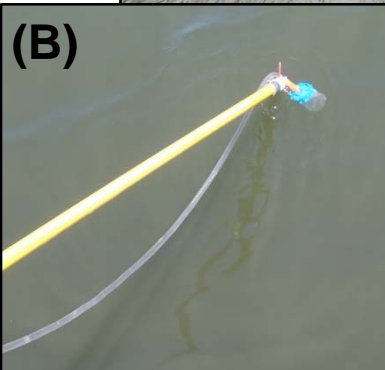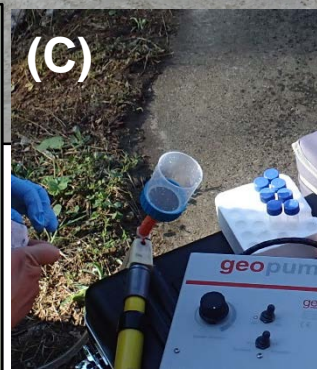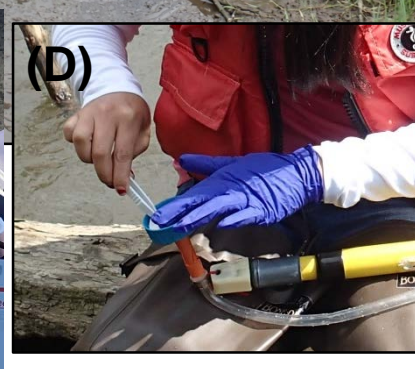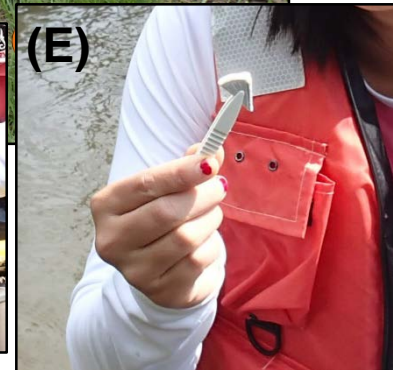

S2\_Fig

Supplement: S2 Fig — (A) Sterile, disposable cup fitted with a glass-fiber filter (1.5um) was attached to a telescoping sampling pole, with tubing was strung along the pole to a peristaltic pump. (B) Sterile cup was placed directly into the water. (C) With this design, only the cup had to be replaced between samples and the pole/exterior of tube rinsed in 10% bleach solution. Filtered water was emptied into graded bucket to keep track of volume filtered. (D) In the field, the filter was removed using sterile technique and (E) placed in an extraction tube; all samples were placed on ice until return to the laboratory. (PDF) [file pone.0191720.s002.pdf]
