# Supplementary material for: Environmental DNA (eDNA): A tool for quantifying the abundant but elusive round goby (Neogobius melanostomus)
Source: PLoS One. 2018 Jan 22;13(1):e0191720. doi: 10.1371/journal.pone.0191720 (PMC5777661; doi:10.1371/journal.pone.0191720)
Supplement: S1 Table — (PDF) [file pone.0191720.s003.pdf]

S1_Table

First author	Year	Species	Decay rate constant (k)
Nevers, MN		Neogobius melanostomus (Roundy Goby), 19°C	0.058
		Neogobius melanostomus (Roundy Goby), 12°C	0.043
Minamoto, T	2017	Chrysaora pacifica (Japanese sea nettle)	0.033
Tsuji, S	2017	Plecoglossus altivelis altivelis (ayu sweetfish)	0.071
		Cyprinus carpio (common carp)	0.074
Sassoubre, LM	2016	Engraulis mordax (Northern Anchovy)	0.101
		Sardinops sagax (Pacific Sardine)	0.068
		Scomber japonicas (Pacific Chub Mackerel)	0.070
		Sardinops sagax (Pacific Sardine) mackerel + sardine	0.057
		Scomber japonicas (Pacific Chub Mackerel) mackerel + sardine	0.055
Maruyama, A	2014	Lepomis macrochirus (bluegill sunfish)	0.051-0.159
Barnes, MA	2014	Cyprinus carpio (common carp)	0.105
Strickler, KM	2014	Lithobates catesbeianus (bullfrog)	0.05-0.34
Thomsen, PF	2012	Platichthys flesus (European flounder)	0.322
		Gasterosteus aculeatus (Three-spine stickleback)	0.701
